# Comparison of the Efficacy of Intra-Articular Polyacrylamide Hydrogel and the Cross-Linked Hyaluronic Acid/Chondroitin Sulfate Combination in Treating Advanced-Stage Knee Osteoarthritis

**DOI:** 10.3390/medicina62061091

**Published:** 2026-06-04

**Authors:** Mustafa Altıntaş, Okan Ateş, Furkan Soy, Tacettin Mirzaoğlu, Mustafa Akif Sarıyıldız

**Affiliations:** 1 Department of Orthopaedics and Traumatology, Gazi Yaşargil Training and Research Hospital, 21010 Diyarbakır, Türkiye; atesokan@msn.com; 2 Department of Orthopedics, Faculty of Medicine, Kırıkkale University, 71450 Kırıkkale, Türkiye; dr.furkansoy@gmail.com; 3 Department Physical Medicine and Rehabilitation, Memorial Dicle Hospital, 21220 Diyarbakır, Türkiye; tacettin.mirzaoglu@memorial.com.tr (T.M.); makifsariyildiz@hotmail.com (M.A.S.)

**Keywords:** Polyacrylamide hydrogel, hyaluronic acid, intra-articular injection, knee, osteoarthritis

## Abstract

*Background and Objectives*: Polyacrylamide hydrogel (PAAG) is still underresearched, and few human studies have validated its efficacy in knee osteoarthritis (OA), particularly in advanced stages. The aim of this study was to compare the efficacy of intra-articular PAAG with that of the widely used cross-linked chondroitin sulfate/hyaluronic acid (HA/CS) combination in treating knee OA in a retrospective trial. *Materials and Methods*: A total of 127 patients diagnosed with grade 3 or 4 knee OA according to the Kellgren–Lawrence scale were included. The first group received an intra-articular injection of the cross-linked HA (60 mg)/CS (90 mg) combination, whereas the second group was administered 6 mL of PAAG. The outcome measures, which were assessed at baseline, 3 months, and 12 months, were knee pain severity measured with the visual analog scale (VAS), range of motion (ROM), the Western Ontario and McMaster Universities Osteoarthritis Index (WOMAC) scale score, and the Pittsburgh Sleep Quality Index (PSQI). *Results*: At three months, the PAAG group demonstrated significantly lower pain and WOMAC scores than the HA/CS group, while ROM and sleep quality scores were not significantly different at either 3 or 12 months. Within-group comparisons revealed significant reductions in pain, WOMAC, and sleep scores in both groups over time, but no significant improvement in ROM was detected in either group. *Conclusions*: Compared with HA/CS injection, intra-articular PAAG injection significantly improved the VAS and WOMAC scores in advanced knee OA patients at three months. However, the outcomes for all the parameters were similar in the two groups at 12 months.

## 1. Introduction

OA is a multifactorial joint disease affecting the entire joint, characterized by cartilage degeneration, subchondral bone remodeling, synovial membrane inflammation, and biomechanical changes in the infrapatellar fat pad [1]. Knee osteoarthritis (OA) is the most common joint disease worldwide and significantly reduces quality of daily life in elderly patients [2,3]. The most important risk factors for osteoarthritis have been reported as weight, age, female gender, joint damage, and muscle weakness [2]. Patients present with progressive pain and loss of function. The incidence of knee OA in society has increased along with average life expectancy [2,3,4]. Current management strategies for knee OA include weight control, isometric exercises, assistive devices, and physical therapy. Pharmacological treatments such as nonsteroidal anti-inflammatory drugs (NSAIDs), topical creams, and intra-articular injections (e.g., hyaluronic acid, platelet-rich plasma [PRP], plasma rich in growth factors [PRGF], and corticosteroids) are effective in treating grades 1 and 2 diseases. However, these treatment modalities often provide only transient benefits or may even be ineffective in patients with grades 3 and 4 diseases [5,6,7].

Hyaluronic acid (HA), one of the compounds most commonly used for intra-articular injections today, has not been shown to differ from other agents regarding short-term results in analyses. However, it has been reported in the literature that its effectiveness regarding therapeutic duration decreases in the long term [8,9]. With the development of regenerative therapies and new intra-articular injections, effective agents have been developed for patients with advanced arthritis, with the most important ones being carboxymethyl-chitosan and polyacrylamide hydrogels (PAAGs) [10]. Polyacrylamide hydrogel (PAAG) has emerged as a promising option for patients and clinicians. Although the acrylamide monomer exhibits carcinogenic and neurotoxic properties, these adverse effects are not observed once the molecule is polymerized [11,12]. PAAG has been used for approximately 25 years in aesthetic procedures and intravesical injections without significant adverse effects. Because this molecule is biocompatible, viscoelastic, and nondegradable [7,11,12], its usability in osteoarthritis has been suggested. PAAG, which had been used in other areas since 2000, was studied for intra-articular injection in horses in 2015, in rabbits and horses in 2016, and in goats in 2017, and later studies, mainly involving lame horses, revealed its effectiveness in treating knee OA [12,13,14]. The first human case series, a 2018 cohort study, included patients with grades 3–4 knee OA (72% of participants) and reported significant improvements in WOMAC scores 13 months post-PAAG injection [15]. Good results for knee intra-articular PAAG injections at a 6-month follow-up were reported in a cohort study conducted three years later [16]. The only randomized controlled trial in the literature, conducted by Bliddal et al., reported findings comparable to those obtained with intra-articular hyaluronic acid injections over a 12-month period, with PAAG not proving to be more effective than HA [17].

Although PAAG remains a novel molecule, few human studies have validated its efficacy in knee OA, particularly in advanced stages. Existing studies are limited by small sample sizes. The effectiveness of PAAG in the treatment of advanced knee OA remains controversial. Therefore, the aim of this study was to compare the efficacy of intra-articular PAAG with that of the established cross-linked HA/CS combination in treating knee OA based on a retrospective design.

## 2. Materials and Methods

### 2.1. Study Design and Patient Characteristics

Demographic data, including age, education level, height, weight, and body mass index (BMI), were recorded. All patients underwent detailed locomotor system examinations, and the knee OA diagnosis was confirmed with clinical evaluation and radiographic imaging (X-ray).

The clinical outcomes of the patients were reviewed using a retrospective design after ethical approval was obtained. This study included patients who underwent intra-articular PAAG or cross-linked HA injections into the knee joint at physical medicine rehabilitation and orthopedic outpatient clinics between March 2023 and April 2024. The inclusion criteria defined patients aged over 55 years with advanced-stage (grades 3–4) knee osteoarthritis according to the Kellgren and Lawrence (KL) classification system [18]. All patients had experienced knee pain that has lasted at least 6 months. The exclusion criteria included BMI > 35, grades 1–2 knee OA, inflammatory rheumatic diseases (e.g., rheumatoid arthritis, Behçet’s disease, and ankylosing spondylitis), hematologic disorders, advanced cardiac insufficiency, current chemotherapy, or intra-articular knee injections (e.g., HA, platelet-rich plasma, and corticosteroids) within the preceding 3 months. Hyaluronic acid (HA/CS) is a widely used and accepted intra-articular treatment method worldwide for viscosupplementation in knee osteoarthritis. Establishing HA/CS as an active control group instead of placebo will both guarantee a broadly beneficial outcome and reveal whether intra-articular PAAG treatment is at least as effective as intra-articular HA. Injections were administered to the more symptomatic knee of each patient. Following the sterilization of the knee with betadine, Group 1 received an intra-articular injection of 60 mg of cross-linked HA (Orthoflex One^®^ S.C. Rompharm Company SRL, Bucharest, Romania) combined with 90 mg of chondroitin sulfate (CS), whereas Group 2 received 6 mL of PAAG (Arthrosamid^®^, Contura International A/S, Copenhagen, Denmark), both in a single injection. The injections used were ready and sterile and were injected by adding only a sterile needle tip. All the injections were administered by a single experienced physician. Postinjection recommendations included cold application for 3 days and daily isometric knee exercises. For this reason, they came to the physiotherapist for weekly check-ups. Both groups were permitted to use NSAIDs for breakthrough pain. The selection of medication to be injected was made at the physician’s discretion, based on the patient’s clinical characteristics and previous treatment history, not in accordance with any predetermined allocation criteria; it was part of routine clinical practice.

This study was reviewed and approved by the Kırıkkale University Faculty of Medicine Ethics Committee (date: 27 May 2024; decision No. 08/2024) and adheres to the tenets of the Declaration of Helsinki. Written informed consent was obtained from all subjects before they were included.

### 2.2. Primary Outcome Measures

Pain severity was measured with a 10 mm visual analog scale (VAS) [19]. Joint swelling, knee range of motion (ROM), the functional score, and sleep quality were assessed at baseline, 3 months, and 12 months. Joint swelling was evaluated manually and with ultrasonography. ROM was quantified with a goniometer during maximal flexion and extension.

### 2.3. Functional Status and Disability

Disease-related functional status and disability were assessed with the Western Ontario and McMaster Universities Osteoarthritis Index (WOMAC) [20]. The WOMAC comprises three subscales: pain (5 items; WOMAC-A), stiffness (2 items; WOMAC-B), and functional limitations (17 items; WOMAC-C). Each item is scored on a 5-point Likert scale (1 = none, 2 = mild, 3 = moderate, 4 = severe, and 5 = extreme), where higher scores indicate greater pain, stiffness, and functional impairment. The Turkish version of the WOMAC was validated by Tüzün et al. [21].

### 2.4. Sleep Quality Assessment

Sleep quality was evaluated via the Pittsburgh Sleep Quality Index, which assesses sleep over the preceding month across 19 items [22]. The index includes seven components: subjective sleep quality, sleep latency, sleep duration, sleep efficiency, sleep disturbances, use of sleep medications, and daytime dysfunction. Each component is scored between 0 and 3 based on symptom frequency, yielding a total score of 0–21; scores of six or higher indicate impaired sleep quality. The purpose of the PSQI is to assess sleep disorders that occur in OA patients, especially those in advanced stages. The Turkish version of the PSQI was validated by Ağargün et al. [23].

### 2.5. Statistical Analysis

The calculations were carried out using Statistical Package for Social Sciences software version 21.0 for Windows (SPSS Inc., Chicago, IL, USA). The Kolmogorov–Smirnov test was used to confirm that the data were normally distributed. Descriptive statistics for continuous variables were expressed as means and standard deviations, and categorical variables were expressed as numbers and percentages. The chi-square test was used to evaluate differences in categorical variables. Student’s *t*-test was used to evaluate significant differences in continuous variables between the two groups. For repeated measures, ANOVA was used to analyze the effects of differences in the scores of disease-related variables at baseline, three months, and twelve months. The Wilcoxon test was used to evaluate the pre- and postinjection differences within the groups. A power analysis of the study and sample size calculation were performed using G*Power version 3.08 software, and the power was calculated as 82% for the primary outcome measures. Statistical significance was based on a value of *p* < 0.05.

## 3. Results

A total of 119 patients (86 males and 33 females; age range: 55–82 years) diagnosed with grade 3 or 4 knee OA according to the KL scale were included in the study. In total, 60 were in the HA group, with 42 female and 18 male patients, and 59 were in the PAAG group, with 44 female and 15 male patients (*p* > 0.05).

Among the 136 knees screened, 9 were excluded because they did not meet the inclusion criteria. Since six of these nine patients had a BMI > 35, the other three patients were excluded due to the previously mentioned comorbid diseases. Therefore, intra-articular injections were administered to 127 knees. Eight patients were excluded during follow-up: five opted for prosthetic surgery (three from the HA/CS group and two from the PAAG group), and three were lost to follow-up. A total of 119 patients (knees) completed the study. In the HA/CS group, 44 knees were classified as grade 3 and 16 as grade 4, whereas in the PAAG group, there were 45 grade 3 and 14 grade 4 knees (*p* > 0.05). There were no statistically significant differences between the groups regarding age, sex, BMI, and education level (*p* > 0.05). The demographic characteristics of both groups are shown in Table 1.

Intergroup comparisons at 3 months revealed significantly lower pain scores (*p* < 0.001) and WOMAC scores (*p* < 0.05) in the PAAG group than in the HA/CS group. However, at 12 months, although pain and WOMAC scores were lower in the PAAG group, the differences were no longer statistically significant (*p* = 0.071 and *p* = 0.142, respectively). The ROM and sleep quality scores were not significantly different at 3 or 12 months (*p* > 0.05; Table 1).

Intragroup analysis (using ANOVA across baseline, 3-month, and 12-month data) revealed similar trends in both groups, which demonstrated significant improvements in pain, WOMAC, and sleep quality scores (*p* <0.001). However, no significant improvement in ROM scores was observed in either group (*p* >0.05; Table 1). When changes between the baseline and 12-month measurements were compared, the PAAG group showed a greater difference, but none of the parameters reached statistical significance (*p* > 0.05) (Table 2).

## 4. Discussion

The findings obtained in the present study demonstrate that intra-articular PAAG, a novel therapeutic agent, provides short-term benefits in pain relief and functional improvement in advanced-stage knee OA, although its long-term efficacy aligns with that of the HA/CS combination.

Intra-articular PAAG injection is a promising and frequently applied treatment option for advanced knee OA. However, the number of studies conducted to date is very limited. Preclinical studies in horses, goats, and rabbits have reported successful outcomes [12,13,14,24] and demonstrated that intra-articular PAAG is non-degradable and biocompatible and that it exerts its effects by integrating into the joint capsule and synovial membrane [12,13]. Histopathological analyses in these models revealed synovial cell proliferation into the gel within 14 days postinjection, followed by angiogenesis and hyperplasia within the inner capsule and synovial membrane [13]. It has also been reported that macrophages phagocytose gel and transform into fibroblast-like synovial cells, thereby establishing a barrier between inflammatory cells and the synovial membrane. This barrier is thought to suppress inflammation and limit intra-articular effusion, suggesting that PAAG injection does not increase joint effusion [13,14]; this has been corroborated in equine studies, which showed reduced effusion over 24 months [14,24]. Although experimental studies in horses generally produced positive results, the average age of lame horses was typically between 2 and 4 years, indicating that these experiments were performed in a very young equine population. In contrast, PAAG is generally used in humans with advanced knee OA, who are typically over 60 years of age. Accordingly, we specifically included patients with advanced knee OA (grades 3–4) in the present study and reported that the long-term effects of the molecule were similar to those of the HA/CS combination.

The first major human study was conducted by Henriksen et al. [15]. Although their cohort included KL grades 1–4, 72% of participants had advanced-stage knee OA (grades 3 and 4). Each patient received a single 6 mL intra-articular PAAG injection, and significant improvements in pain and functional scores were observed at months 7 and 13. Bliddal et al. investigated PAAG efficacy in patients with grade 2–4 knee OA over a 12-month follow-up and reported significant reductions in pain and WOMAC scores [11]. A 3- and 5-year follow-up study conducted by the same study group confirmed sustained improvements in pain and function [25,26]. In another single-arm study, although patients showed significant improvements regarding pain and functions after 24 months, 18 percent required total knee replacement [27]. However, none of these studies included a control group. Similarly, in the present study, the PAAG group presented lower pain and functional scores than the HA/CS group at 12 months, but compared with the HA/CS group, no statistically significant differences were detected, and the HA/CS group demonstrated comparable improvements. In a randomized controlled trial by Bliddal et al. [17], 239 patients with grade 2–4 knee OA were followed for 12 months (HA: *n* = 120; PAAG: *n* = 119). Although the PAAG group showed numerically better performance across all pain and WOMAC subscores, no statistically significant differences were observed between the groups. The same research group showed that the effectiveness of PAAG continued after 5 years, resulting in a 16.2% improvement in pain scores compared with baseline scores [28]. These findings align with our results. The aim of the present study was to measure PAAG performance specifically in patients with advanced knee OA. All patients were grades 3–4, which may account for the diminished long-term efficacy of PAAG. In a retrospective study by Aykaç et al., the effectiveness of PAAG, steroids, and hyaluronic acid was examined; although PAAG was partially more effective regarding pain and functionality in the first 6 months, no significant difference was observed among the groups at the 12th month [29].

No serious adverse events occurred in our cohort, and no patient experienced increased joint effusion postinjection. In the PAAG group, three patients reported mild pain and fullness for five days post-procedure. Overgaard et al. retrospectively reviewed 91 patients to assess adverse events; a total of 15 patients reported transient distension, but none experienced serious side effects such as infection or ROM restriction. To date, no evidence of PAAG toxicity has been reported in the literature [30,31].

In the meta-analyses conducted, the greatest problem with HA, ozone therapy, and other injections is the short duration of treatment [32,33]. Carboxymethyl-chitosan (CM-C), a new molecule similar to PAAG, is a newly used agent in advanced OA treatment. Manocchio et al. reported a significant decrease in pain in the short and medium term; however, a gradual decrease in effectiveness by the 12th month compared with the 6th month was observed during the treatment period, similar to HA [10]. PAAG has been presented as a good alternative due to its longer-term effect. Although our study showed better results in the short term, we found no significant differences in the one-year results between HA and PAAG. PAAG treatment may provide good outcomes for patients who do not require surgery or whose general condition does not permit surgery. Longer-term follow-up studies should be planned to provide clearer information on this molecule with the aim of reducing the need for arthroplasty in patients with knee osteoarthritis.

Our study had several important limitations. The primary limitation was its retrospective nature; the retrospective design increases the risk of selection bias, which also limits control over confounding factors. Second, although we followed our cases for 12 months, the long-term outcomes of PAAG remain uncertain. Additionally, the sample size was relatively limited. Third, the inclusion of advanced-stage OA cases may have limited the efficacy of PAAG. Advanced-stage, longer-term, prospective, randomized controlled studies are needed to provide more definitive, objective outcomes the role of PAAG in knee OA. When considering the effectiveness of PAAG treatment in moderate and advanced OA, specific subgroup analyses are needed to more objectively determine the treatment response.

## 5. Conclusions

Compared with HA/CS injection, intra-articular PAAG injection significantly improved the VAS and WOMAC scores of patients with advanced knee OA at 3 months. Although the long-term efficacy of PAAG is similar to that of HA, PAAG may be a promising treatment option for elderly patients or those with comorbidities for whom surgery is not recommended. However, long-term randomized controlled trials are needed to better understand the efficacy of PAAG.

## Figures and Tables

**Table 1:** Intragroup (*p* ^1^) and intergroup (*p* ^2^) comparison of demographic and clinical variables.

Patient Characteristics	HA/CS Group (*n* = 60) Mean ± SD	*p* ^1^ *ANOVA*	PAAG Group (n = 59) Mean ± SD	*p* ^1 ^ *ANOVA*	*p* ^2^
Age	67.43 ± 7.42		69.55 ± 8.74		0.156
Gender (F/M)	42/18		44/15		0.346
BMI	30.65 ± 3.32		31.50 ± 4.47		0.232
Radiological grade (KL) 3/4	44/16		45/14		0.262
***Pain severity*** baseline ^VAS^	8.89 ± 0.55		8.93 ± 0.63		0.715
3 months ^VAS^	6.88 ± 1.47	<0.001	4.85 ± 2.03	<0.001	<0.001
12 months ^VAS^	7.18 ± 1.01		6.80 ± 1.74		0.142
***ROM*** baseline	116.75 ± 11.45		114.91 ± 12.92		0.412
3 months	118.50 ± 11.37	0.086	115.50 ± 12.78	0.712	0.176
12 months	117.25 ± 11.11		114.23 ± 13.70		0.188
***WOMAC total*** baseline	58.16 ± 8.94		60.15 ± 9.14		0.158
3 months	45.12 ± 11.12	<0.001	40.65 ± 9.04	<0.001	0.021
12 months	56.43 ± 11.40		55.30 ± 12.10		0.583
***Sleep quality*** baseline	34.45 ± 4.67		35.45 ± 7.53		0.400
3 months	27.65 ± 4.23	<0.001	28.55 ± 7.65	<0.001	0.427
12 months	33.25 ± 4.15		33.31 ± 6.89		0.950

F: female; M: male; KL: Kellgren–Lawrence; BMI: body mass index; VAS: visual analog scale; ROM: range of motion; WOMAC: Western Ontario and Mc Master University Osteoarthritis Index; *p* ^1^: intragroup comparison (ANOVA); *p* ^2^: intergroup comparison (Student’s *t*-test).

**Table 2:** Change scores of primary outcomes from baseline to 12 months.

Patient Variables	HA/CS Group (*n* = 60) Mean ± SD	PAAG Group (*n* = 59) Mean ± SD	*p*
Pain severity	1.71 ± 1.06	2.13 ± 1.75	0.145
ROM	1.50 ± 2.23	1.7 ± 1.23	0.565
WOMAC	1.72 ± 1.07	4.85 ± 2.45	0.075
Sleep quality	1.20 ± 0.90	2.10 ± 0.78	0.439

ROM: range of motion; WOMAC: Western Ontario and Mc Master University Osteoarthritis Index.

## Data Availability

The data presented in this study are available upon request from the corresponding author. The data are not publicly available due to legal and ethical considerations.

## Abbreviations

The following abbreviations are used in this manuscript:

PAAG	Polyacrylamide hydrogel
HA/CS	Chondroitin sulfate/hyaluronic acid
OA	Osteoarthritis
BMI	Body mass index
ROM	Range of motion
PSQI	Pittsburgh Sleep Quality Index
NSAIDs	Nonsteroidal anti-inflammatory drugs
PRP	Platelet-rich plasma
WOMAC	Western Ontario and McMaster Universities Osteoarthritis Index
KL	Kellgren–Lawrence
VAS	Visual analog scale
